# Staphylococcus aureus Tolerance and Genomic Response to Photodynamic Inactivation

**DOI:** 10.1128/mSphere.00762-20

**Published:** 2021-01-06

**Authors:** Sara B. Snell, Ann Lindley Gill, Constantine G. Haidaris, Thomas H. Foster, Timothy M. Baran, Steven R. Gill

**Affiliations:** a Department of Microbiology and Immunology, University of Rochester Medical Center, Rochester, New York, USA; b Center for Oral Biology, University of Rochester Medical Center, Rochester, New York, USA; c Department of Imaging Sciences, University of Rochester Medical Center, Rochester, New York, USA; University of Nebraska Medical Center

**Keywords:** photodynamic inactivation, tolerance, PDI transcriptome, genomic adaptation, PDI tolerance

## Abstract

Staphylococcus aureus is an opportunistic pathogen with a clinical spectrum ranging from asymptomatic skin colonization to invasive infections. While traditional antibiotic therapies can be effective against S. aureus, the increasing prevalence of antibiotic-resistant strains results in treatment failures and high mortality rates. Photodynamic inactivation (PDI) is an innovative and promising alternative to antibiotics. While progress has been made in our understanding of the bacterial response to PDI, major gaps remain in our knowledge of PDI tolerance, the global cellular response, and adaptive genomic mutations acquired as a result of PDI. To address these gaps, S. aureus HG003 and isogenic mutants with mutations in *agr*, *mutS*, *mutL*, and *mutY* exposed to single or multiple doses of PDI were assessed for survival and tolerance and examined by global transcriptome and genome analyses to identify regulatory and genetic adaptations that contribute to tolerance. Pathways in inorganic ion transport, oxidative response, DNA replication recombination and repair, and cell wall and membrane biogenesis were identified in a global cellular response to PDI. Tolerance to PDI was associated with superoxide dismutase and the S. aureus global methylhydroquinone (MHQ)-quinone transcriptome network. Genome analysis of PDI-tolerant HG003 identified a nonsynonymous mutation in the quinone binding domain of the transcriptional repressor QsrR, which mediates quinone sensing and oxidant response. Acquisition of a heritable QsrR mutation through repeated PDI treatment demonstrates selective adaption of S. aureus to PDI. PDI tolerance of a *qsrR* gene deletion in HG003 confirmed that QsrR regulates the S. aureus response to PDI.

**IMPORTANCE** Staphylococcus aureus can cause disease at most body sites, with illness ranging from asymptomatic infection to death. The increasing prevalence of antibiotic-resistant strains results in treatment failures and high mortality rates. S. aureus acquires resistance to antibiotics through multiple mechanisms, often by genetic variation that alters antimicrobial targets. Photodynamic inactivation (PDI), which employs a combination of a nontoxic dye and low-intensity visible light, is a promising alternative to antibiotics that effectively eradicates S. aureus in human infections when antibiotics are no longer effective. In this study, we demonstrate that repeated exposure to PDI results in resistance of S. aureus to further PDI treatment and identify the underlying bacterial mechanisms that contribute to resistance. This work supports further analysis of these mechanisms and refinement of this novel technology as an adjunctive treatment for S. aureus infections.

## Abstract (precis)

Staphylococcus aureus can cause disease at most body sites, with illness ranging from asymptomatic infection to death. The increasing prevalence of antibiotic-resistant strains results in treatment failures and high mortality rates. S. aureus acquires resistance to antibiotics through multiple mechanisms, often by genetic variation that alters antimicrobial targets.

## INTRODUCTION

Staphylococcus aureus is an opportunistic pathogen with a clinical spectrum ranging from asymptomatic skin colonization to invasive infections that are often prolonged and severe (1, 2). Evolutionary adaptations lead to antimicrobial resistance and result in multidrug-resistant S. aureus strains that are difficult to eradicate and account for nearly half of all deaths associated with antibiotic-resistant pathogens (3). S. aureus acquires resistance to antibiotics through multiple mechanisms, including horizontal transfer of antibiotic-modifying enzymes, expression of efflux pumps, and metabolic adaptation to nocuous selection pressures, and by accumulation of genome mutations that alter antimicrobial targets and cell wall composition (4, 5). Introduction of genetic variation by spontaneous mutations is a major driver of bacterial evolution, with the survival and spread of variant strains being dependent on their adaptation to the host environment and selection by antimicrobial therapy (6, 7). In S. aureus and other pathogens, hypermutators with reduced replication fidelity and higher mutation rates emerge during chronic infections, when the ability to adapt quickly can be beneficial for evading the host immune response and antibiotic therapy (8). The response and adaptation of S. aureus hypermutators to antibiotic therapy are illustrated by the *in vivo* evolution of vancomycin-susceptible S. aureus to vancomycin-intermediate S. aureus (VISA), a polygenic trait which develops through multiple mutations in the genome that are associated with increased susceptibility to host innate immunity and reduced susceptibility to antibiotics (9, 10). As a result of this evolutionary capacity and the limited development of new antibiotics, the prevalence of multidrug-resistant S. aureus strains in health care and community settings is rapidly reducing treatment options for staphylococcal diseases.

A promising alternative to antibiotics, photodynamic inactivation (PDI), employs a photosensitizer (PS) that accumulates in the target bacteria and becomes activated from a ground to an excited state when exposed to visible light (11–14). This excited photosensitizer can undergo electron transfer to form several reactive oxygen species (type I mechanism) or can react directly to molecular oxygen to form a highly reactive singlet oxygen (type II mechanism) (11). The resulting oxidation and functional damage to multiple cellular targets, including membrane lipids, proteins, and nucleic acids, result in inactivation of essential cellular functions and bacterial death (11, 12). PDI has been shown to effectively inactivate a diverse array of bacterial and fungal pathogens, in *in vitro* studies with planktonic cells, under *in vivo* conditions using murine models with infected burn or excisions wounds, and in human clinical studies on oral dental plaque (14). Because PDI is nonselective and affects multiple cellular targets, development of tolerance to PDI has been considered to be unlikely (13, 15), and until the recent work by Rapacka-Zdonczyk et al. (13), attempts to induce S. aureus tolerance upon repeated sublethal doses of PDI had not succeeded (16, 17). Using repeated sublethal doses of PDI, Rapacka-Zdonczyk et al. (13) generated clinical isolates of S. aureus and other Gram-positive bacteria that were tolerant to PDI and further demonstrated that the development of tolerance was a result of an increased DNA mutation rate with upregulation of error-prone DNA polymerase V (*umuC*) and induction of the SOS response (13). However, the authors did not examine potential global changes in S. aureus metabolism, membrane transporters, gene regulation, cellular structure, or heritable gene mutations that may contribute to the PDI tolerance phenotype.

In this study, we sought to identify the mechanisms that contribute to PDI tolerance in S. aureus. The S. aureus strain HG003 (18) and isogenic HG003 mutants with mutations in DNA methyl mismatch repair (*mutSL*) and the accessory gene regulatory network (*agr*) (8) were used to evaluate the response of S. aureus to repeated sublethal doses of PDI. Global transcriptome and genome analyses were used in an agnostic approach to identify the underlying regulatory and genetic adaptations that occur as a result of repeated PDI and contribute to PDI tolerance. Our results demonstrate that repeated sublethal doses of PDI lead to selective evolution of PDI tolerant strains, with a global transcriptional response in numerous cellular functions and acquisition of a heritable mutation in the quinone-sensing transcriptional regulator, QsrR.

## RESULTS

### PDI of S. aureus using the photosensitizer MB.

**FIG 1:**
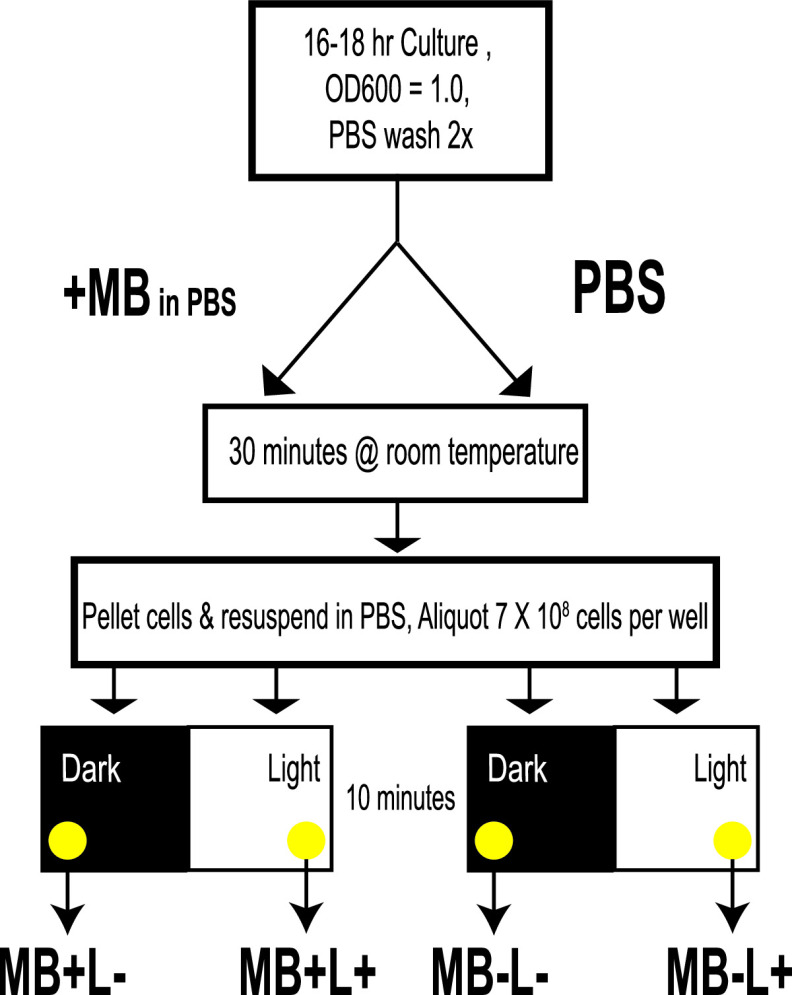
Schematic representation of PDI and control treatments. The diagram summarizes a single or initial treatment with MB+L+ (PDI), MB+L−, MB−L+, and MB−L−. After 10 min of light or dark exposure, cells are serially diluted and plated for CFU counting. For each sequential treatment, fresh TSB is inoculated directly from each of the four different treatment groups and cultured to stationary phase, and treatment is repeated. A separate culture is inoculated from a single colony on a TSA plate to serve as the PDI-naive control.

**FIG 2:**
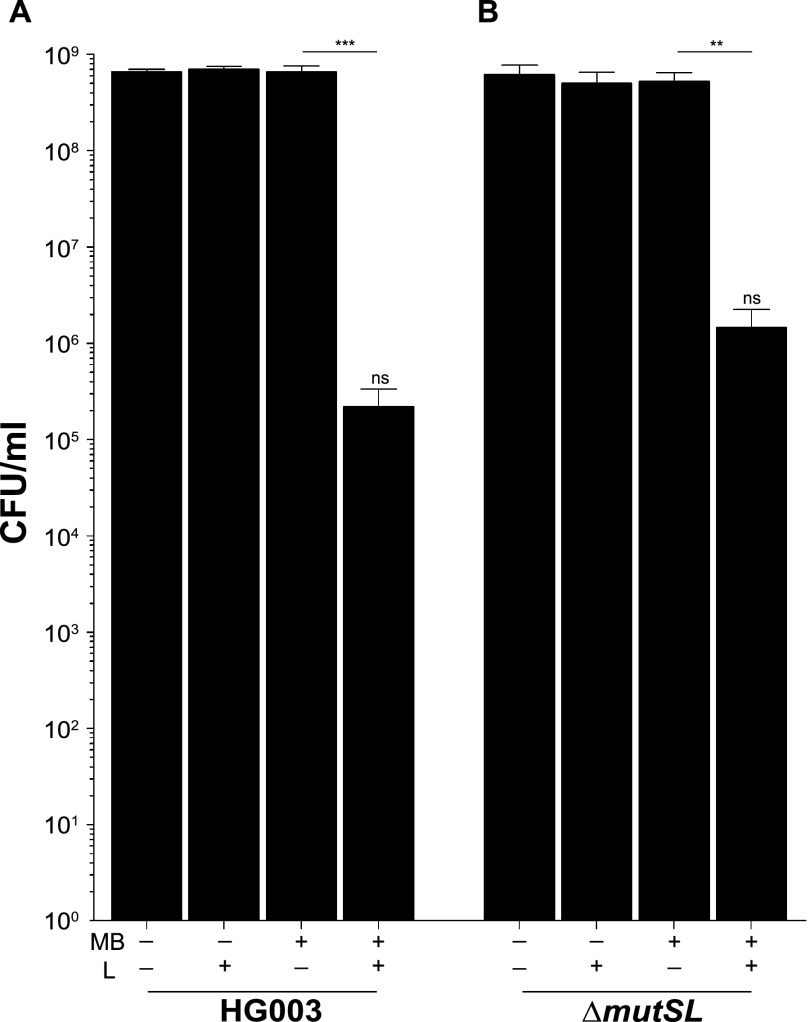
There is no difference in PDI susceptibility between parental and hypermutator strains. PDI was performed on S. aureus strains HG003 (A) and HG003Δ*mutSL* (B). Data were collected from three independent experiments performed in duplicate. Error bars represent standard deviations (SD). Statistical differences were determined by an unpaired, two-tailed *t* test. ***, *P* = 0.004 (HG003 MB+L− versus MB+L+); **, *P* = 0.002 (HG003Δ*mutSL* MB+L− versus MB+L+); ns, not significant (*P* = 0.556) (HG003 MB+L+ versus HG003Δ*mutSL* MB+L+).

Methylene blue (MB) is an effective photosensitizer when used in PDI against both methicillin-sensitive and methicillin-resistant S. aureus (MSSA and MRSA) growing as planktonic cells (19) and in biofilms (20). We chose MB as a photosensitizer because it is approved for use in a variety of clinical settings (21). In the present study, stationary-phase cultures of S. aureus strain HG003 were treated with 33 μM MB for 30 min, at which point cells were washed and resuspended in phosphate-buffered saline (PBS) to remove excess dye (Fig. 1). Cells were then exposed to broadband visible light for 10 min, which corresponded to a fluence of 2.4 J cm^−2^, and plated on tryptic soy agar (TSA) for enumeration of surviving CFU. Under these conditions, there was a 3- to 4-log reduction in CFU in HG003 that received MB treatment and were exposed to light (MB+L+) compared to controls that either did not receive MB and were shielded from light (MB−L−), did not receive the MB treatment but were exposed to light (MB−L+), or received MB but were shielded from light (MB+L−) (Fig. 2A). Under these conditions there was no dark toxicity of the MB and no effects of light on CFU formation. These were the standard conditions used throughout this study for both initial and repeated PDI treatment. In this report, the term “PDI” refers only to MB+L+ treatment.

### HG003 MMR and GO DNA repair mutants (Δ*mutSL* and Δ*mutY* mutants) have susceptibility to PDI similar to that of HG003.

**FIG 3:**
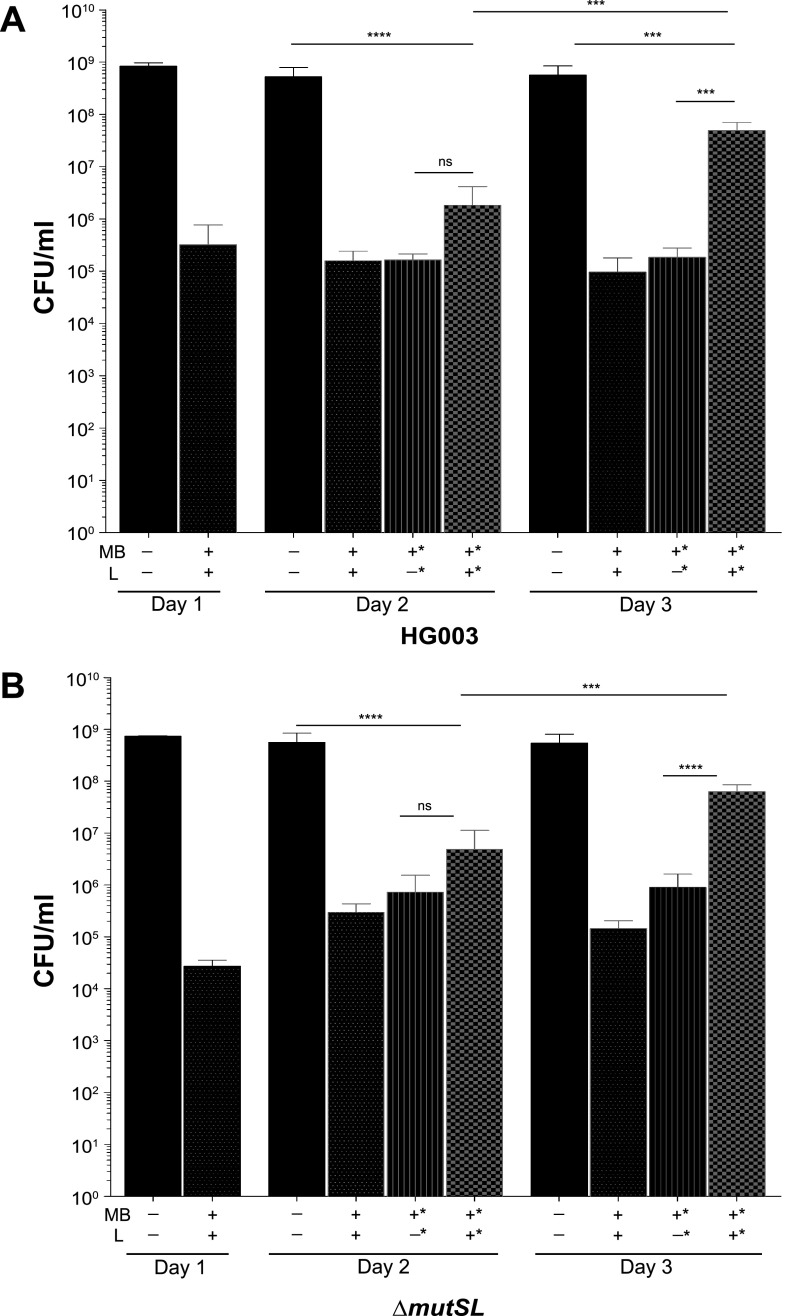
Repeated exposure to PDI results in tolerance in HG003 and HG003Δ*mutSL*. After initial PDI treatment of (A) HG003 and (B) HG003Δ*mutSL*, aliquots of each treatment group (MB−L−, MB−L+ MB+L−, and MB+L+) were inoculated into fresh TSB and cultured for a subsequent round of PDI. Additionally, each day a naive culture of S. aureus was inoculated from a single colony on TSA as an unpassaged control. This procedure was repeated sequentially for three successive days. Data were collected from three independent experiments performed in duplicate. Asterisks in the *x* axis labels denote sequentially treated groups. Error bars represent SD. Statistical differences were determined by an unpaired, two-tailed Student's *t* test. ****, *P* = 0.0003 (HG003 day 3 MB+L+ versus MB+L−); ***, *P* < 0.0001 (HG003Δ*mutS* day 3 MB+L+ versus MB+L−); ns, not significant.

To examine the potential contribution of S. aureus mismatch repair (MMR) DNA repair mechanisms to PDI susceptibility and development of PDI tolerance, we compared the PDI response of HG003 to that of an isogenic MMR mutant, HG003Δ*mutSL*, that we previously constructed to examine phenotypic responses of S. aureus hypermutators (8). When treated with the same PDI conditions as wild-type HG003 (MB+L+), HG003Δ*mutSL* (MB+L+) exhibited a reduction in CFU similar to that of controls (MB−L−, MB−L+, and MB+L−) (Fig. 2B). To evaluate the ability of HG003 and HG003Δ*mutSL* to adapt to the selective pressure of repeated PDI exposure, cells (1.75 × 10^7^ CFU) surviving treatment were inoculated into fresh medium and grown overnight. Cells were then exposed to the same treatment conditions the following day. This procedure was repeated for 3 days, by which time both HG003 and HG003Δ*mutSL* receiving PDI treatment (MB+L+) exhibited increased survival upon repeat exposure compared to naive and MB+L− sequentially treated controls (Fig. 3A and B). We also examined a potential role for DNA repair by the oxidized-guanine (GO) system in susceptibility to PDI. We hypothesized that because guanosine is susceptible to oxidation by PDI-associated reactive oxygen species (ROS), a GO mutant would be susceptible to PDI. However, survival of a GO mutant strain, HG003Δ*mutY*, was similar to that of the parental HG003 strain (data not shown). Therefore, development of PDI tolerance cannot be attributed to a hypermutator phenotype previously demonstrated with these strains (8). We note that development of tolerance to PDI in this and subsequent experiments is likely due to our experimental approach, in which suspensions of 1.75 × 10^7^ CFU from each exposure to PDI were used for the subsequent passage in each of the repeated exposures. Unlike previous studies, which passaged a single colony for each exposure (16), our approach increases the probability that the majority of the surviving or tolerant population in each exposure is carried forward into the subsequent exposure.

### Repeat exposure of S. aureus HG003 and ATCC 25923 results in PDI tolerance.

**FIG 4:**
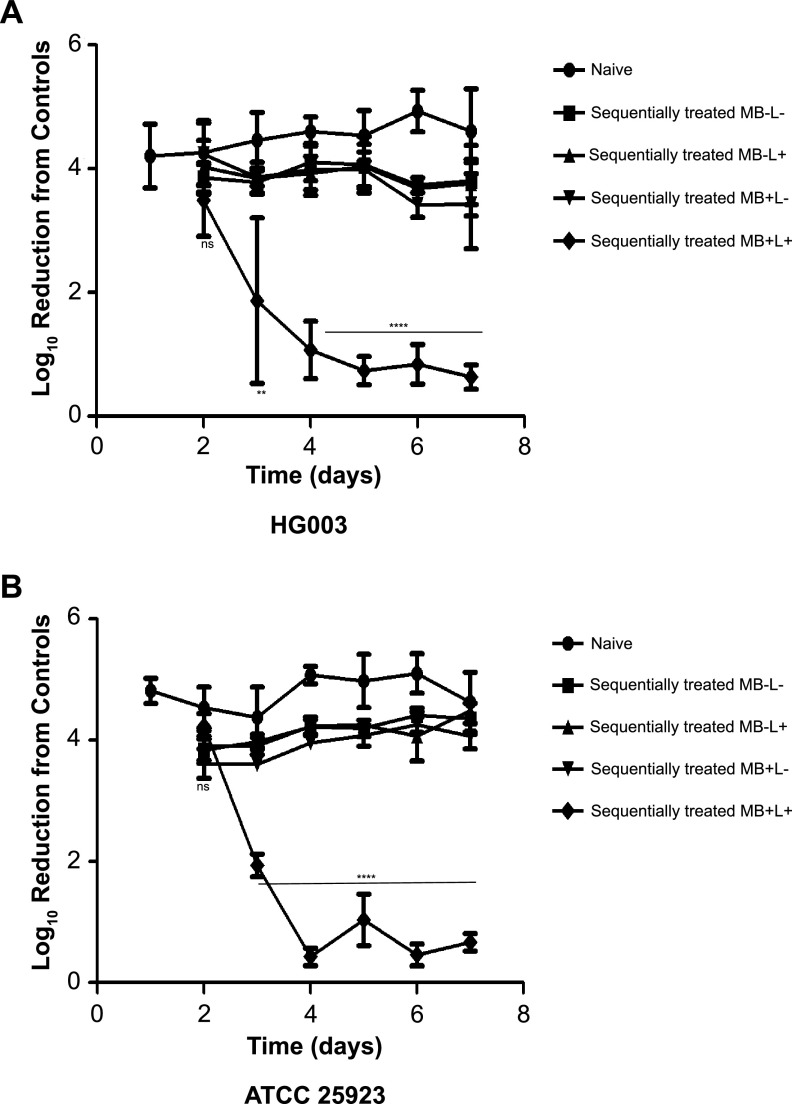
Repeated exposure to PDI results in enhanced resistance in S. aureus strains HG003 and ATCC 25923. After initial PDI treatment of (A) HG003 or (B) ATCC 25923, aliquots of each treatment group (MB−L−, MB−L+ MB+L−, and MB+L+) were inoculated into fresh TSB and cultured for a subsequent round of treatment under the same four conditions. Additionally, each day a naive culture of S. aureus was inoculated from a single colony on TSA as an unpassaged control. This procedure was repeated sequentially for seven successive days. Results are expressed as a decrease in log_10_ CFU reduction from the controls. Data were collected from three independent experiments performed in duplicate. Error bars represent SD. Statistical differences were determined by a two-way analysis of variance (ANOVA) followed by Bonferroni's posttest comparing the naive treatment group. ****, *P* < 0.0001.

We evaluated the development of tolerance or increased survival to PDI by HG003 and compared the response of HG003 to ATCC 25923, a strain previously shown not to develop PDI tolerance upon repeated exposure to MB and broadband visible light (22). After a 7-day sequential exposure of PDI of both HG003 and ATCC 25923, MB+L+-treated cells exhibited a level of tolerance to PDI upon repeat exposure similar to that of naive and 7-day sequentially treated controls (MB−L−, MB−L+, and MB+L−) (Fig. 4). The level of tolerance, expressed as a decrease in log_10_ reduction relative to the controls, increased significantly from the initial PDI treatment through day 4 and then remained constant through day 7. To test whether HG003 isolates that developed tolerance would withstand PDI at a higher concentration of MB, we increased the concentration used on the 7-day MB+L+ PDI-resistant population from 33 to 66 μM. The increase in MB to 66 μM resulted in a 1-log reduction in HG003 compared to 33 μM MB (see Fig. S1 in the supplemental material). Although this decrease was not statistically significant, the results suggest that the concentration of MB in PDI is a factor in tolerance.

10.1128/mSphere.00762-20.1 FIG S1 PDI of resistant cells with 2× MB concentration. Four repeat exposures to PDI were performed on HG003*agr*::*tetM*. Results are expressed as log_10_ reduction from the controls. Data were collected from 3 independent experiments performed in duplicate. Error bars represent SD. Statistical differences were determined by an unpaired, two-tailed Student’s *t* test (**, *P* = 0.0035). Download FIG S1, PDF file, 0.07 MB. Copyright © 2021 Snell et al. 2021 Snell et al. This content is distributed under the terms of the Creative Commons Attribution 4.0 International license.

### Quantitation of MB associated with naive and PDI sequentially treated cells.

**FIG 5:**
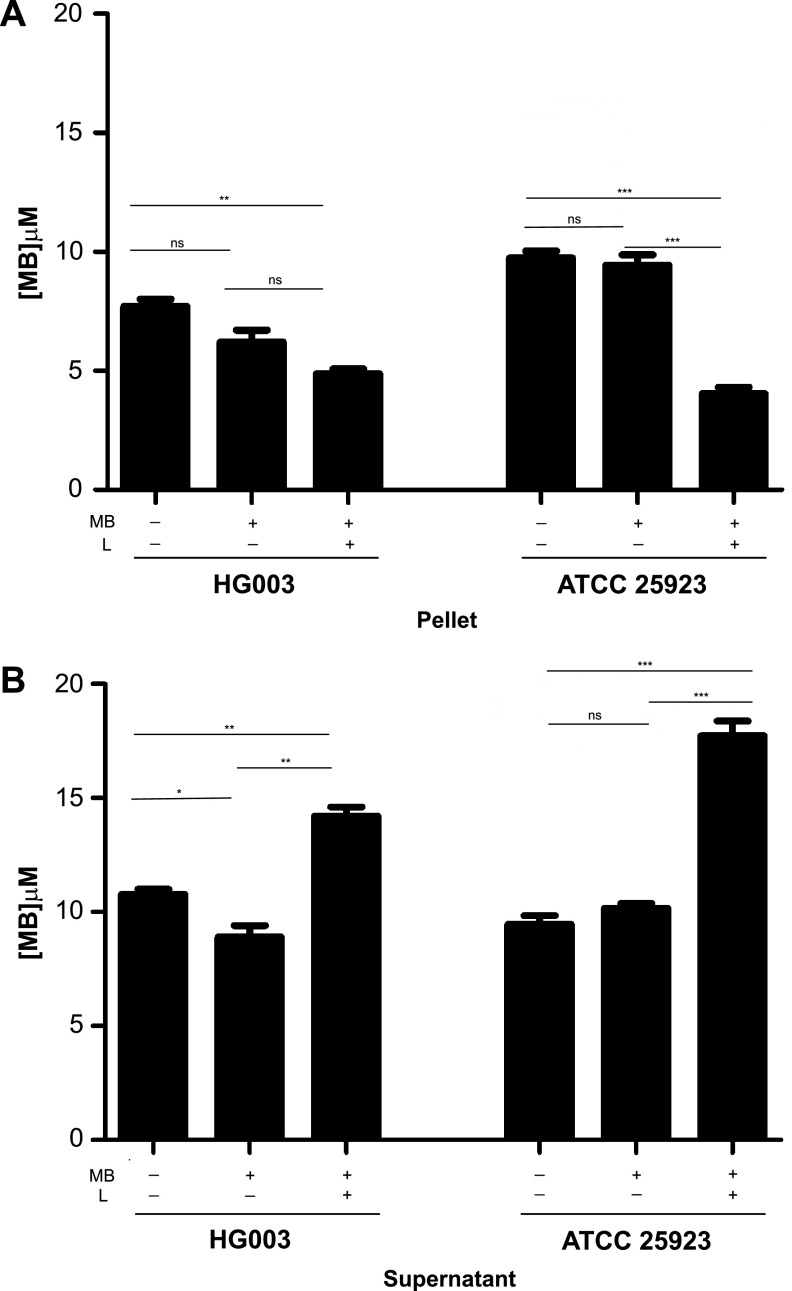
Quantitation of MB associated with PDI-tolerant cell pellets. Cultures of naive bacteria, bacteria exposed to seven sequential daily MB+L+ treatments, and bacteria exposed to seven sequential daily MB+L− treatments were treated with 12.5 μg/ml (33 μM) MB at room temperature for 30 min. After incubation, cells were pelleted and aliquots of the supernatant were saved for MB quantification. Cell pellets were resuspended in an equal volume of PBS. MB was quantitated for both the cell pellet-associated suspension (A) and supernatant (B) using an absorption reading measured spectrophotometrically. Data were collected from 3 independent experiments performed in duplicate. Error bars represent SD. Statistical differences were determined by an unpaired, two-tailed Student's *t* test. *, *P* < 0.03; **, *P* < 0.002; ***, *P* < 0.0004; ns, not significant.

Following the washing step of the repeat-passage PDI experiments, we observed consistent differences in the color of the cell pellet between naive cells and PDI-passaged cells. Because this observation suggested that PDI tolerance developed as a result of a decrease in MB bound by bacteria, we measured absorbance to quantitate the amount of MB associated with the bacterial cell pellets and supernatants of each treatment group. The amount of MB associated with the pellet of sequentially treated PDI (MB+L+) cells was significantly less than that measured for the naive cells (Fig. 5A) and corresponded inversely to the amount of MB remaining in the supernatant of each treatment group (Fig. 5B). These results suggest that the sequentially PDI-treated cells do not convert MB to its colorless leuco-reduced form, a colorless, photochemically inactive form of MB (23). Because the decreased concentration of MB associated with the cell pellets was not due to leuco-reduction, it suggests that the MB either was not retained by the cells or was actively extruded by efflux pumps or other cell wall transporters (15).

### Effect of MB removal by PBS washing prior to PDI.

The PBS wash to remove excess MB is not consistently applied in PDI studies, with some studies bypassing the washing step prior to broadband light exposure (24, 25). To exclude washing as a factor that contributed to development of PDI tolerance in our study, we performed a 4-day repeat exposure but without the PBS wash prior to broadband light exposure. While S. aureus HG003 treated in this way maintained the ability to develop tolerance (Fig. S2), the tolerance developed more quickly and was 2-fold lower for the unwashed than the washed HG003 at the day 4 repeat exposure (compare Fig. S2 with Fig. 4).

10.1128/mSphere.00762-20.2 FIG S2 PDI resistance study without a washing step. Four-day repeat exposures to PDI without the washing step were performed on HG003. Data were collected from 3 independent experiments performed in duplicate. Error bars represent SD. Statistical differences were determined by a two-way ANOVA followed by Bonferroni's posttest comparing to the naive treatment group. ****, *P* < 0.0001. Download FIG S2, PDF file, 0.06 MB. Copyright © 2021 Snell et al. 2021 Snell et al. This content is distributed under the terms of the Creative Commons Attribution 4.0 International license.

### Cells treated sequentially with PDI using MB are also more tolerant to PDI using a structurally similar photosensitizer, TBO.

**FIG 6:**
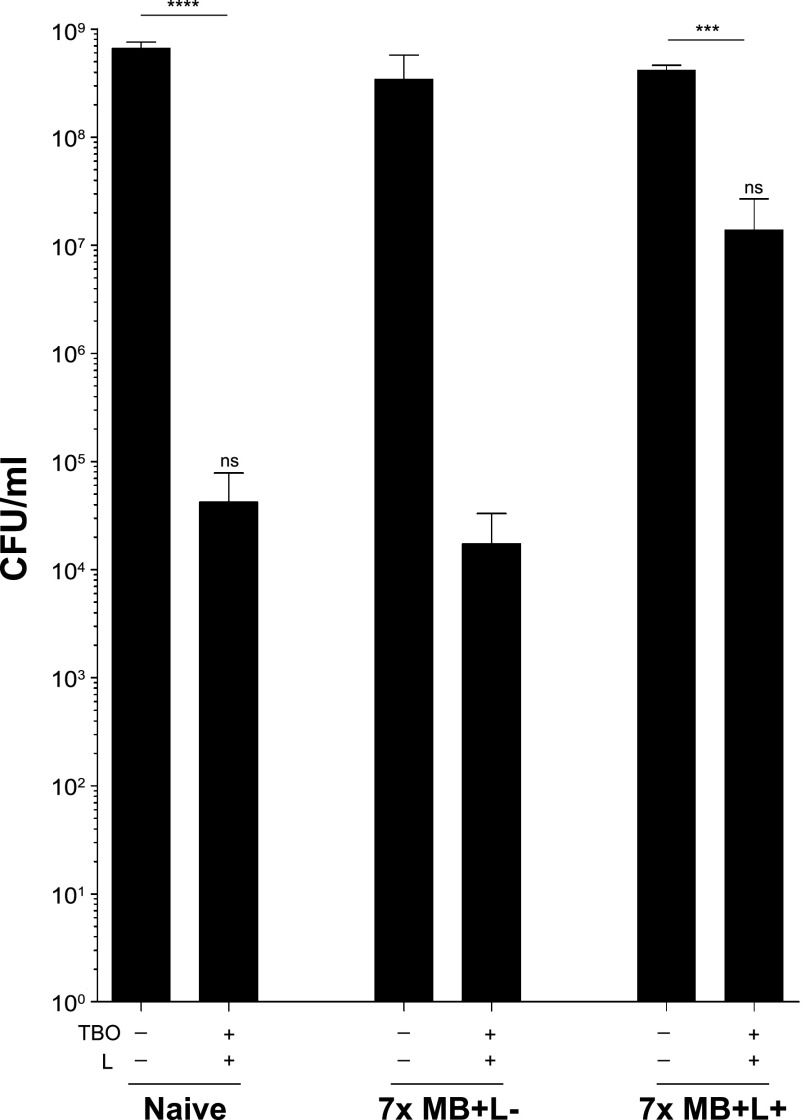
PDI-tolerant S. aureus are tolerant to PDI treatment with toluidine blue O. Cultures of naive bacteria, bacteria exposed to seven sequential daily MB+L+ treatments, and bacteria exposed to seven sequential daily MB+L− treatments were subjected to a final PDI using TBO instead of MB (TBO+L+). Data were collected from 3 independent experiments performed in duplicate. Error bars represent SD. Statistical differences were determined by an unpaired, two-tailed Student's *t* test. ***, *P* < 0.013 (controls versus treated); ****, *P* < 0.004 (controls versus treated); ns, not significant (*P* > 0.05) (naive versus MB+L+ treated).

To evaluate whether the PDI tolerance phenotype seen with MB+L+-passaged cells would be conferred to cells treated with toluidine blue O (TBO), a photosensitizer structurally similar to MB, 7-day MB+L− and MB+L+ sequentially treated cells were subjected to a final PDI using 10.2 μM TBO instead of MB (TBO+L+). This concentration of TBO was determined by titration, as it resulted in a log reduction of naive cells comparable to that achieved with 33 μM MB. The MB+L+ sequentially treated cells exhibited a less dramatic reduction in CFU than the naive or MB+L− sequentially treated cells compared to controls after treatment with TBO+L+, showing similar tolerance phenotypes with both photosensitizers (Fig. 6).

### The S. aureus gene *agr* does not contribute to PDI tolerance.

Previous studies have shown conflicting results regarding the role of *agr* (accessory gene regulator) in PDI susceptibility (15, 26–28). The results appear to be dependent on the genotype of the S. aureus strains and structure of photosensitizers used for PDI. To evaluate the ability of HG003*agr*::*tetM* to adapt to a selective pressure of repeated PDI exposure, cells surviving an initial PDI treatment were inoculated into fresh medium and grown overnight. After a 3-day sequential exposure of HG003*agr*::*tetM* to MB+L+, the cells exhibited a level of tolerance to PDI upon repeat exposure similar to that of naive and sequentially treated controls (MB−L−, MB−L+, and MB+L−) (Fig. S3). This outcome demonstrates that *agr* does not contribute to PDI susceptibility or tolerance.

10.1128/mSphere.00762-20.3 FIG S3 PDI resistance develops in S. aureus HG003*agr*::*tetM*. Four repeat exposures to PDI were performed on HG003*agr*::*tetM*. Results are expressed as log_10_ reduction from the controls. Data were collected from 3 independent experiments performed in duplicate. Error bars represent SD. Statistical differences were determined by a two-way ANOVA followed by a Bonferroni's posttest comparing to the naive treatment group. ****, *P* < 0.0001. Download FIG S3, PDF file, 0.07 MB. Copyright © 2021 Snell et al. 2021 Snell et al. This content is distributed under the terms of the Creative Commons Attribution 4.0 International license.

### Increased tolerance to PDI is maintained in cells passaged in the absence of PDI.

**FIG 7:**
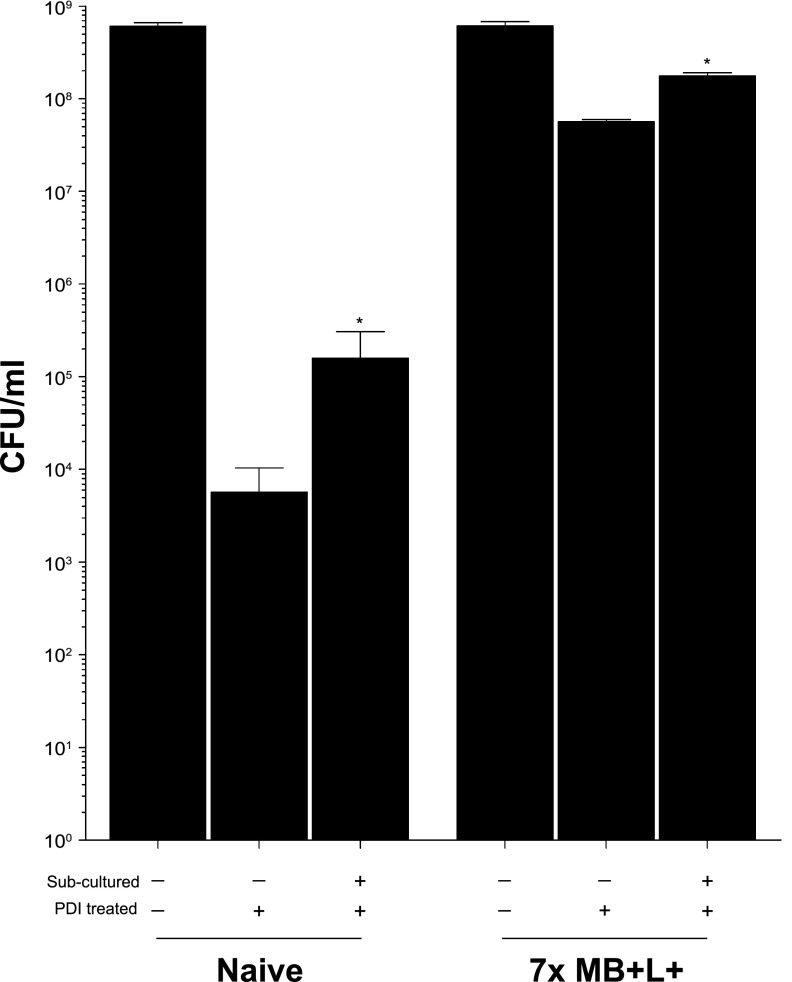
Resistance to PDI is maintained in cells passaged in the absence of PDI. Naive bacteria and bacteria exposed to seven sequential daily MB+L+ treatments were cultured without treatment in TSB for 24 h. These cultures were then diluted 1:200 in fresh TSB and grown overnight. This subculture step was repeated over three sequential days. On the third day, the fresh naive and 7-day MB+L+ cultures were inoculated in parallel with the 3-day subculture groups. PDI was then performed as in previous experiments. Data were collected from 3 independent experiments performed in duplicate. Error bars represent SD. Statistical differences were determined by an unpaired, two-tailed Student's *t* test. *, *P* = 0.0405 (naive subculture treated versus MB+L+ subcultured treated).

To distinguish whether the tolerance phenotype was a transient physiological adaptation or stable and genetically heritable, we passaged PDI-tolerant cells in the absence of selective pressure for 3 days by subculturing daily into fresh medium and then performed PDI. While subcultured naive cells had a slightly elevated tolerance to PDI compared to naive cells that had not been subcultured, both groups were more sensitive to PDI than the MB+L+ sequentially treated cells. PDI-tolerant cells passaged in the absence of selective pressure (MB+L+ subculture positive) maintained a level of resistance similar to that of cells that were not subcultured (MB+L+ subculture negative) (Fig. 7). This outcome suggests that PDI tolerance is stable and may be the result of heritable mutations in HG003 that affect function or expression of genes that control the response to PDI.

### Transcriptional analysis of PDI-treated and PDI-tolerant S. aureus HG003.

**FIG 8:**
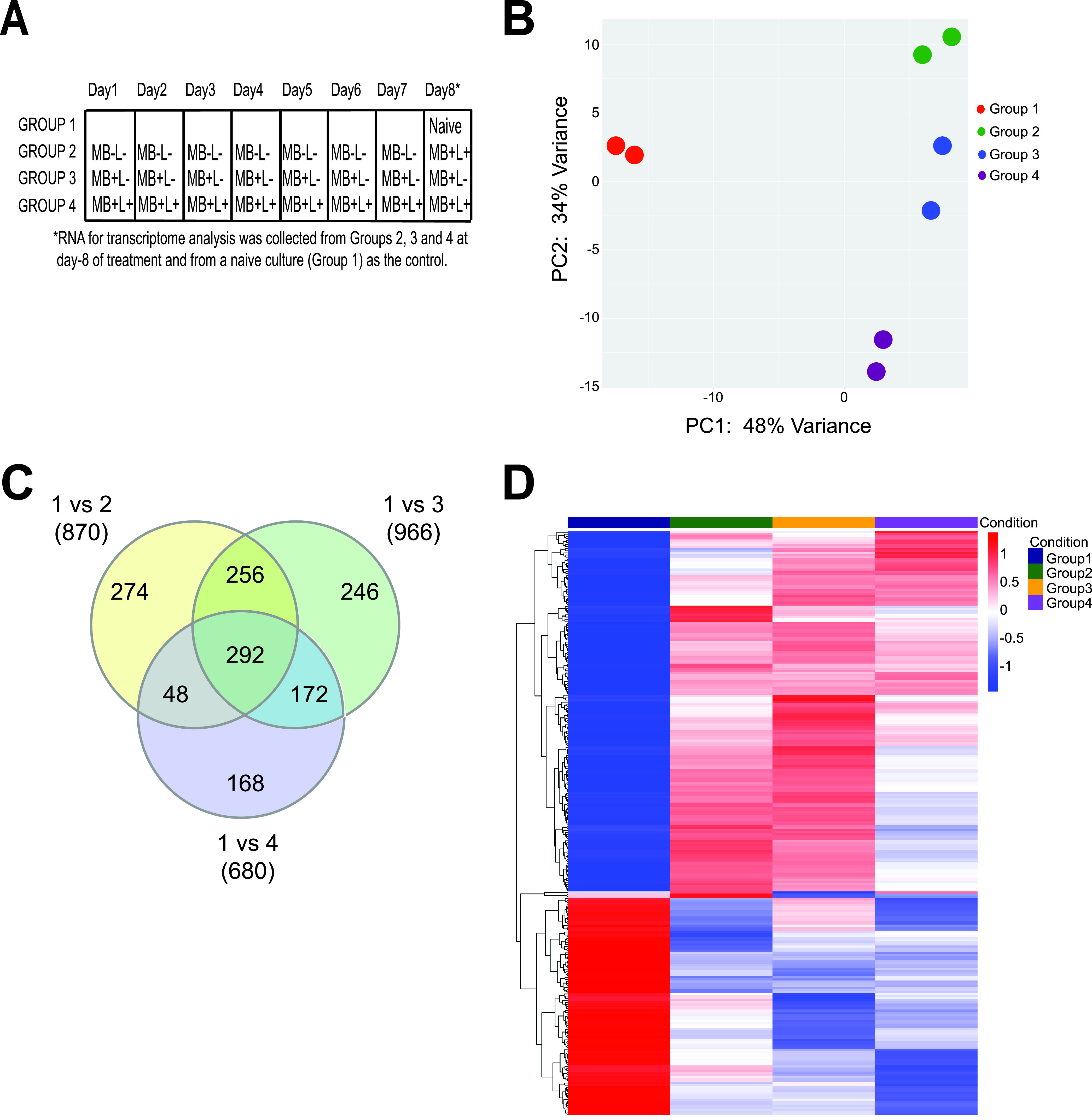
Transcriptional analysis of PDI-treated and PDI-tolerant S. aureus HG003 revealed the regulatory and genetic adaptations that contribute to tolerance. (A) RNA-Seq was used to identify S. aureus HG003 genes that were differentially regulated in response to seven sequential daily MB−L− treatments followed by a single MB+L+ (PDI) treatment (group 2), seven sequential daily MB+L− treatments followed by a single MB+L− treatment (group 3), and seven sequential daily MB+L+ treatments followed by a single MB+L+ (PDI) treatment (group 4). Naive S. aureus HG003 that was not subcultured or exposed to MB−L−, MB+L−, or MB+L+ treatments (group 1) served as a baseline for expression to which all other treatment groups were compared. RNA-Seq sequencing reads were mapped to the S. aureus NCTC8325 genome and differential gene expression determined with a 95% confidence interval and a false discovery rate (FDR) of 0.05. A greater-than-log_2_-fold increase or decrease in expression level was used to identify genes expressed at significantly different levels between experimental groups. A *P* value cutoff of 0.05 was set to identify genes that were significantly different in expression between group 1 and groups 2, 3, and 4. Results of two independent replicates are shown in the remaining panels. (B) Principal-component analysis (PCA) of PDI treatment groups. PCA was performed using the plotPCA function from the DESeq2_1.10.1 package and normalized counts per million reads for 1,140 available genes. (C) Venn diagram of differentially expressed genes in PDI treatment groups. The Venn diagram was generated using genes that were differentially expressed in each treatment group compared to no treatment. (D) Heat map of 292 differentially expressed genes in common between groups 2, 3, and 4. Genes were hierarchically clustered based on Euclidean distance of the row-scaled data with complete linkage.

We next used RNA-Seq to identify S. aureus genes that were differentially regulated in response to the following treatment regimens: seven sequential daily MB−L− treatments followed by a single MB+L+ (PDI) treatment (group 2), seven sequential daily MB+L− treatments followed by a single MB+L− treatment (group 3), and seven sequential daily MB+L+ treatments followed by a single MB+L+ (PDI) treatment (group 4). Naive S. aureus HG003 that was not subcultured or exposed to MB−L−, MB+L−, or MB+L+ treatments (group 1) served as a baseline for expression to which all other treatment groups were compared (Fig. 8A). The group 2 population is PDI susceptible, and the group 4 population is PDI tolerant. Genes that were differentially expressed (greater than a log_2_ fold change at a false discovery rate [FDR] of 0.05 and a *P* value of <0.05) in groups 2, 3, and 4 relative to the untreated bacteria (group 1) are listed in Table S2 in the supplemental material. Differential expression between selected genes in both capsular biosynthesis and inorganic ion transport and metabolism were validated by qRT-PCR analysis (Fig. S4).

10.1128/mSphere.00762-20.4 FIG S4 qRT-PCR indicates similar trends in differential expression of select genes. Average relative copy number was calculated by qRT-PCR analysis in capsular biosynthesis pathway genes (A) and inorganic ion transport and metabolism genes (B). Data represent two replicates performed in triplicate, with error bars representing SD. Statistical differences were determined by an unpaired, two-tailed Student’s *t* test. ****, *P* < 0.0001; ***, *P* = 0.0002; **, *P* = 0.0024. NCTC8325 gene loci are as follows: SAOUHSC_00122, Cap5I; SAOUHSC_00123, Cap5J; SAOUHSC_00124, Cap5K; SAOUHSC_00325, EfeO; SAOUHSC_00748, FeABC; and SAOUHSC_02428, HtsB. Download FIG S4, PDF file, 0.2 MB. Copyright © 2021 Snell et al. 2021 Snell et al. This content is distributed under the terms of the Creative Commons Attribution 4.0 International license.

Principal-component analysis of the differentially expressed pathways and abundance in each group revealed that the four treatment groups formed distinct clusters, with biological replicates tending to group together within those clusters (Fig. 8B). A total of 870 genes in group 2, 996 genes in group 3, and 680 genes in group 4 were differentially expressed compared to those in group 1 (Fig. 8C). We conclude that genes found in common for each of these comparisons (292 genes) represent transcriptional changes in response to treatment with MB alone and no light. We also conclude that the 168 genes unique to group 4 are associated with the tolerance phenotype—or that tolerance is a result of changes in these 168 genes plus the 292 found in common among these comparisons. Heat maps of hierarchically clustered genes common to all treatment groups (Fig. 8D) demonstrated that the functional groups with the largest number of differentially expressed genes, both those common to the PDI-tolerant group (single MB+L+ PDI treatment group [group 2]) and those unique to the PDI-tolerant group (8-day sequential MB+L+ treatment group [group 4]), encompass a broad range of functions, including amino acid transport and metabolism, inorganic ion transport and metabolism, replication, regulation, recombination and repair, and cell wall and membrane biogenesis. All significant differentially regulated genes from groups 2, 3, and 4 are listed by group, pathway, and function in Table S2. This broad response reflects the nonselective nature of PDI, oxidative damage to multiple cellular targets, and the inactivation of essential cellular functions.

We first focused on differential gene expression in four functional categories among groups 2, 3, and 4: response to oxidative stress, detoxification of reactive electrophiles, DNA repair mechanisms, and cell wall surface proteins and structures. Enzyme genes that contribute to detoxification and response to oxidative stress that were differentially regulated collectively among the three groups included SAOUHSC_00320, which encodes a NADH-dependent flavin mononucleotide reductase; SAOUHSC_00173, which encodes a flavin mononucleotide (FMN)-dependent NADH-azoreductase; SAOUHSC_00833, which encodes a nitroreductase family protein; SAOUHSC_02825, which encodes a glyoxalase family protein; SAOUHSC_00318, which encodes a glyoxalase/bleomycin resistance protein; and SAOUHSC_00093, which encodes manganese-dependent superoxide dismutase (29, 30). Differentially regulated genes that contribute to DNA recombination and repair mechanisms included SOS response proteins (LexA, SAOUHSC_01333; RecJ, SAOUHSC_01744; RecN, SAOUHSC_01615; RecO, SAOUHSC_01667; RecR, SAOUHSC_00445; and RadC, SAOUHSC_01763) and DNA mismatch repair proteins (MutS, SAOUHSC_01272; MutL, SAOUHSC_01273; MutT, SAOUHSC_00429; and MutY, SAOUHSC_02005). A number of cell wall and membrane protein, inorganic ion transporter, and metabolism related genes were differentially regulated in common between groups 2, 3, and 4, suggesting that this is a result of interaction of methylene blue with the bacterial surface and transport into S. aureus and is independent of light (Table S2). Common among these three groups was the downregulation of SAOUHSC_00122 (Cap5I), SAOUHSC_00123 (Cap5J), and SAOUHSC_00124 (Cap5K), the three genes in the capsular 5 biosynthesis pathway that are responsible for translocation of the final precursor lipids to the outer cell surface, where they polymerize to form a protective polysaccharide microcapsule on the S. aureus surface (31). Unique to group 3 was downregulation of SAOUHSC_02877, encoding dehydrosqualene desaturase (CrtN), which together with SAOUHSC_2789, encoding dehydrosqualene synthase (CrtM), forms the biosynthetic pathway for S. aureus carotenoids which protects S. aureus against killing by ROS (32, 33).

We next focused on genes that were differentially expressed only in the PDI-tolerant population, with the view that these genes are required for the tolerant phenotype and that their response is due to a heritable mutation in the S. aureus HG003 genome (Table S2). Genes that were upregulated in the PDI-tolerant population included those for enzymes that contribute to detoxification and response to oxidative stress described above (FMN-dependent NADH-azoreductase, nitroreductase, and superoxide dismutase), the iron transport siderophores SirA (SAOUHSC_00074), SirB (SAOUHSC_00072), HtsA (SAOUHSC_02430), HtsB (SAOUHSC_02428), and HtsC (SAOUHSC_02427), and global transcriptional regulators, including MgrA (SAOUHSC_00694) and SaeS (SAOUHSC_00714). The *sirAB* genes have been implicated in the detoxification of ROS, as deletion mutants have been shown to be more sensitive to H_2_O_2_ and have reduced survival in macrophages (31). MgrA and SaeS regulate expression of multiple S. aureus virulence factors and multidrug efflux pumps and have also been found to be involved in the response to oxidative stress (26, 29, 34).

### Genomic analysis and identification of nucleotide mutations in PDI-susceptible and PDI-tolerant S. aureus HG003.

**FIG 9:**
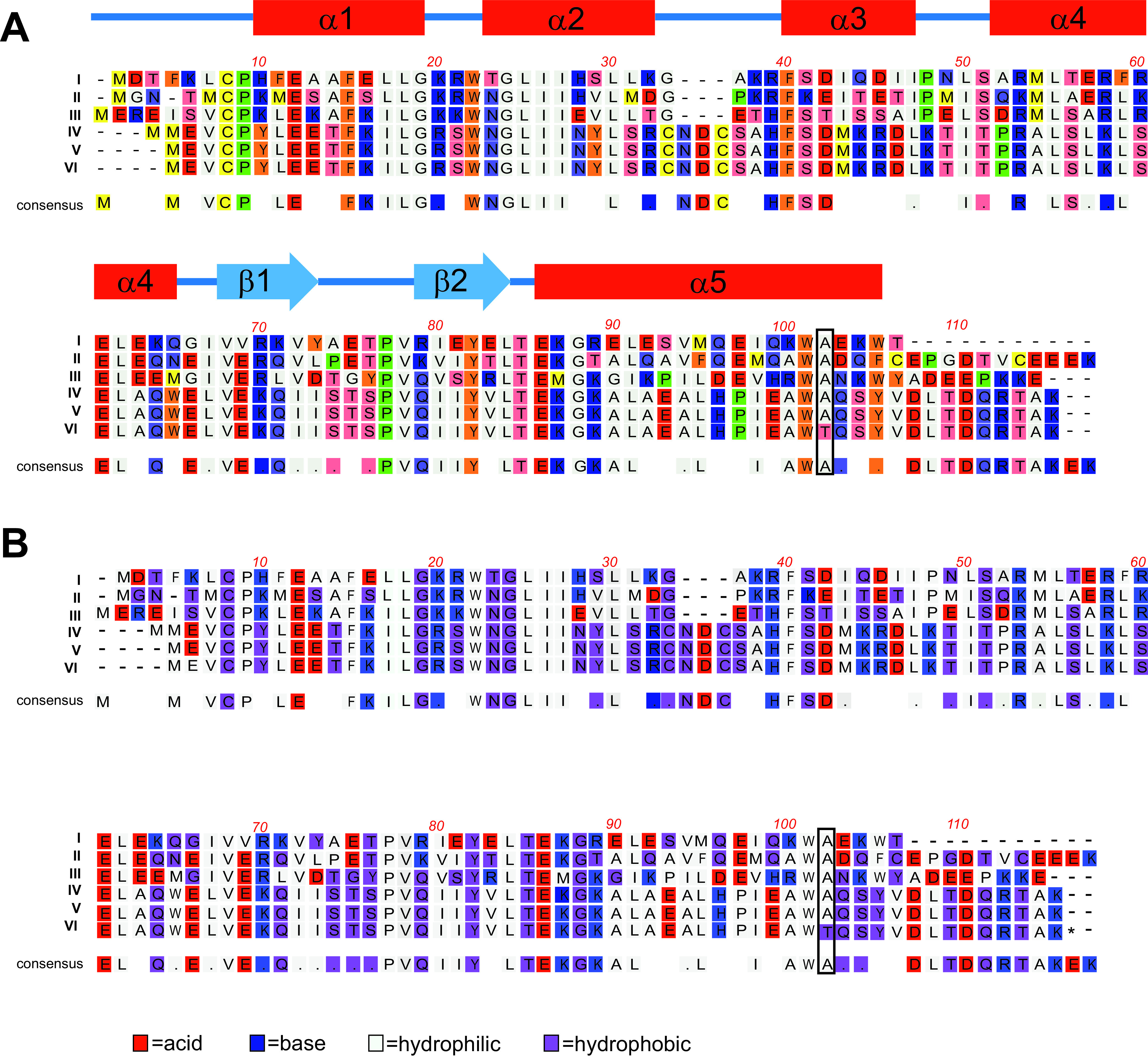
Sequence alignment of five members of the YodB/QsrR family and the S. aureus HG003 PDI-tolerant mutant. Protein sequences of YodB/QsrR from the following bacteria are aligned as I to VI as follows: Clostridium (GenBank no. WP_010964167; I), *Bacillus subtilis* (GenBank no. NP_389835; II), *Sporolactobacillus inulinus* (Genbank no. WP_010027434; III), *Staphylococcus aureus* (GenBank no. 4HQE_A; IV), S. aureus NCTC8325 (GenBank no. ABD31395.1; V), and S. aureus HG003 (PDI tolerant; VI) were aligned using MacVector Clustal W. Conserved amino acids within the YodB/QsrR family are included as a consensus sequence. A period indicates conservation between groups of weakly similar properties, scoring ≤0.5 in the Gonnet PAM 250 matrix. (A) Amino acids are color coded by chemical and the secondary structure of QsrR, indicated above the sequence with helices in red and sheets in blue. The transition from a C to a T at nucleotide 2186246 in the genome of the PDI-tolerant strain (Table S3) results in the change from a nonpolar alanine to a polar threonine at amino acid 98 in the α5′ helices at the dynamic interface of the quinone binding pocket. The position of this amino acid change is indicated by the box at amino acid 98 of QsrR from the S. aureus strains and amino acid 102 in the consensus alignment of all included members of the YodB/QsrR family. (B) The same alignment with amino acids color coded by function, indicating a shift from hydrophilic to hydrophobic at the mutation.

To determine if S. aureus acquired nucleotide mutations as a result of PDI treatment and if these mutations contributed to the observed PDI tolerant phenotype, we completed whole-genome sequencing of S. aureus HG003 that was not passaged or exposed to PDI and isolates from groups 1 (MB−L−), 3 (MB+L−), and 4 (MB+L+) at days 1, 3, and 7 of sequential treatment. The genomic data are summarized in Table S3, with sequenced isolates designated by treatment groups (G1, G2, and G3) and sequential days of treatment (D1, D3, and D7). The S. aureus NCTC8325 genome sequence served as the reference genome to which the genomes from untreated HG003 and all treatment groups were compared. As expected, we identified known genomic variants (single nucleotide polymorphisms [SNPs]) in SAOUHSC_02301 (*rsbU*) and SAOUHSC_02636 (*tcaR*) of untreated HG003 that were repaired when HG003 was derived from NCTC8325. We anticipated that these two SNPs and the additional 33 SNP variants identified between NCTC8325 and HG003 G1D1 in our analysis would be retained in genomes from all other treatment groups and that additional SNP variants would be identified in day 7 PDI treatment group 4, which is PDI tolerant. Two additional SNPs were identified, one in an intergenic region in groups 1, 3, and 4. A second SNP variant, found only in the PDI-tolerant strain, was identified in the QsrR transcriptional regulator (SAOUHSC_02364), a quinone-sensing and oxidant response regulator. The nonsynonymous SNP replaces a nonpolar alanine with a polar threonine at amino acid 98 in the α5′ helices at the dynamic interface of the quinone binding pocket (Fig. 9). Acquisition of this heritable mutation in QsrR as a result of repeated PDI treatment suggests evolutionary adaption of S. aureus to PDI.

### Identification of QsrR as a response regulator of PDI.

**FIG 10:**
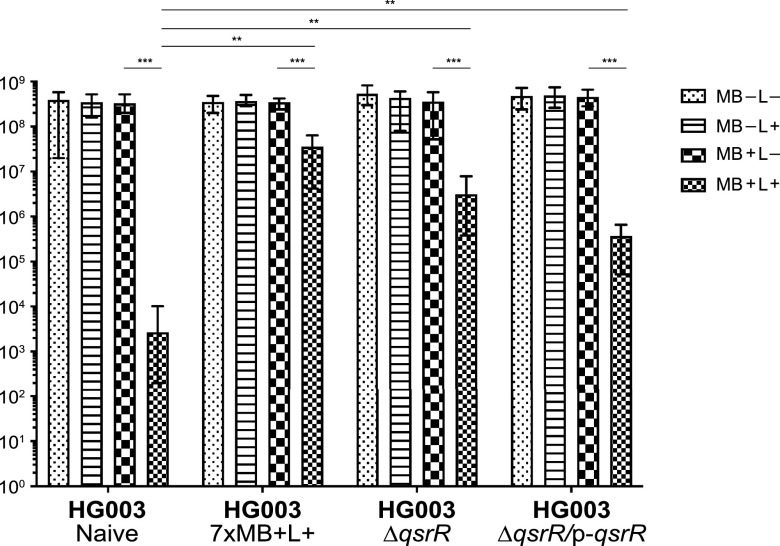
Tolerance to PDI is regulated by the quinone-sensing transcriptional regulator, QsrR. Naive HG003, naive HG003 with a deletion of *qsrR* (HG003Δ*qsrR*), naive HG003 with a *qsrR* deletion complemented with the full-length *qsrR* gene (HG003Δ*qsrR* p-*qsrR*), and a 7-day sequentially MB+L+-treated HG003 strain were cultured in TSB for 24 h. The cells were washed twice in PBS, adjusted to an OD_600_ of 1.0 in PBS (approximately 7 × 10^8^ CFU/ml), and treated with MB−L−, MB−L+, MB+L−, and MB+L+ as described in Materials and Methods (“Initial PDI treatment”). Data were collected from 3 independent experiments performed in duplicate. Error bars represent SD. Statistical differences were determined by an unpaired, two-tailed *t* test. **, *P* < 0.003 (controls versus treated); ***, *P* < 0.00003 (naive treated versus other MB+L+ treated).

To confirm that the nonsynonymous SNP in *qsrR* directly contributed to PDI resistance, we compared the effect of PDI treatment on *qsrR* gene deletion and complemented HG003 strains (HG003Δ*qsrR* and HG003Δ*qsrR* p-*qsrR*) to naive HG003 and resistant (7× MB+L+) strains (Fig. 10). After treatment with MB+L+, HG003Δ*qsrR* exhibited PDI resistance that was increased relative to that of naive HG003 (*P* < 0.003) and similar to that of the 7× MB+L+ population. The *qsrR*-complemented strain exhibited a decrease in resistance, but not to the same level as the naive wild-type HG003 strain. The partial recovery of the PDI-sensitive phenotype when complemented with p-*qsrR* is similar to that observed by Ji et al. (30) in their characterization of the QsrR repressor, suggesting that deletion of *qsrR* affects additional functions that are not recovered by complementation. Overall, these data demonstrate that QsrR regulates the response of S. aureus to PDI.

## DISCUSSION

Staphylococcus aureus is an opportunistic pathogen that impacts humans in both community and hospital settings, where it is capable of severe and invasive infections. While traditional antibiotic therapies can be effective against S. aureus, the increasing prevalence of antibiotic-resistant strains results in treatment failures and high mortality rates. Photodynamic inactivation (PDI) is an innovative nonantibiotic approach using photosensitizers activated by visible light, resulting in the production of cytotoxic reactive oxygen species. While much progress has been made in our understanding of the bacterial response to PDI, major gaps remain in our knowledge of resistance or tolerance to PDI, the global cellular response of pathogens to PDI, and potential adaptive evolution through genomic mutations acquired as a result of PDI. To address these gaps, we used global transcriptome and genome analyses in an agnostic approach to identify the underlying regulatory and genetic adaptations that occur as a result of repeated PDI treatment and that contribute to PDI tolerance. The results of our study reveal the multiple pathways in amino acid transport and metabolism, inorganic ion transport, DNA replication recombination and repair, and cell wall and membrane biogenesis that are associated with PDI tolerance. Whole-genome analysis of PDI-tolerant HG003 isolates identified a nonsynonymous mutation in the quinone binding domain of the quinone-sensing transcriptional regulator, QsrR, which mediates stress sensing and response to oxidants (29, 30). Acquisition of this heritable QsrR mutation as a result of repeated PDI treatment suggests selective adaption of S. aureus to PDI.

We first asked if repeated exposure to sublethal PDI treatment in S. aureus would lead to PDI-tolerant populations and if tolerance is more likely to emerge in hypermutator strains with defective DNA mismatch repair (MMR). Unexpectedly, there was no significant difference in PDI susceptibility (Fig. 2) or PDI tolerance (Fig. 3A and B) between S. aureus HG003 and the HG003Δ*mutSL* hypermutator strain. These results suggest that MMR does not have a direct role in the PDI response, as has been shown with *umuC*, which encodes a stress-responsive error-prone DNA polymerase V in the SOS response regulatory network (13, 35). However, the increased expression of *mutS* and *mutL* in the 7-day sequentially treated groups 2, 3, and 4 (Table S2) suggests an undefined role for MMR in the PDI response. The lack of widespread and deleterious genome mutations in the PDI-treated and tolerant populations relative to the untreated bacteria (Table S3) demonstrates active DNA repair in the PDI response. We note that these experimental approaches measure two distinct responses of the bacterial population to PDI. Transcriptome analysis assesses the immediate response to PDI with RNA extracted from mixed populations of bacteria immediately after treatment. As a result, these data represent bacteria that are either dead, dying, recovering from PDI, or tolerant to PDI. In contrast, whole-genome sequencing analysis samples only the subpopulation of bacteria that remain viable after treatment and are cultured prior to extraction of genomic DNA. In other words, for the bacteria that receive a single PDI treatment, where there is a low level of survival, the transcriptome identifies the responses of all bacteria, while WGS reveals genomic diversity only in the bacteria that survive. In the PDI-tolerant population, where the majority of bacteria survive, the transcriptome and WGS reflect the responses and genomic changes in the surviving population.

The broad transcriptional responses common to all PDI treatment groups illustrate the far-ranging impact of oxidative damage on numerous essential cellular functions. All significant differentially regulated genes from groups 2 (PDI susceptible), 3 (MB+ only), and 4 (PDI tolerant) are listed by group, pathway, and function in Table S2. While there are four distinct clusters of transcriptome responses (Fig. 8B and D and Table S2), many of the functional changes are shared within these groups. This broad response reflects the nonselective nature of PDI, oxidative damage to multiple cellular targets, and the inactivation of essential cellular functions. The response in the PDI-tolerant population suggests that tolerance is partially dependent on increased expression of the MgrA regulon as well as the sensor histidine protein kinase SaeS, which was previously shown by Gandara et al. (26) to impair the response to PDI. Expression of *mgrA* and *saeS* was increased only in the PDI-tolerant population, not those subjected to a single exposure of MB+L+ or multiple exposures of MB+L−. The MgrA, SaeSR, and SAOUHSC_02364 (QsrR) regulons as well as SAOUHSC_02322, encoding the CsoR repressor, which is downregulated in the PDI-tolerant population (Table S2), fall within the S. aureus global methylhydroquinone (MHQ)-quinone transcriptome network that confers resistance to quinones, which form highly reactive semiquinone radicals that promote generation of ROS and superoxide anions (36). QsrR contributes to quinone detoxification by controlling expression of antioxidant enzymes in the Qsr regulon, including NADH-dependent flavin mononucleotide reductase, nitroreductase family protein, glyoxalase family protein, and a glyoxalase/bleomycin resistance protein encoded by SAOUHSC_00318, as well as riboflavin biosynthesis genes (SAOUHSC_01886, SAOUHSC_01887, SAOUHSC_01888, and SAOUHSC_01889) (29, 30, 36, 37). Riboflavin is the central component of the factors flavin adenine dinucleotide (FAD) and FMN and required by all flavoenzymes which function in the reduction and detoxification of quinones (30). Expression of these genes in the QsrR regulon and riboflavin genes was increased in our PDI-tolerant population, which substantiates its role in the PDI quinone-oxidative response.

As a transcriptional repressor, QsrR regulates expression through direct binding of the QsrR dimer to the gene promoter region, where it represses expression of quinone detoxification systems. Sensing and binding of menadione/quinone to the binding pocket formed by the QsrR dimer cause conformational change of the DNA-bound QsrR, leading to release of QsrR from the promoter and activation of the quinone regulon detoxification systems. The acquisition of a nonsynonymous SNP resulting in replacement of a nonpolar alanine with a polar threonine at amino acid 98 (Fig. 9) in the α5′ helices at the dynamic interface of the menadione/quinone binding pocket raises the possibility that a structural change of the binding pocket will prevent binding of QsrR to promoters of genes in the Qsr regulon in the PDI-tolerant population, resulting in constitutive increased expression of the Qsr regulon. Increased PDI tolerance of a *qsrR* gene deletion strain (HG003Δ*qsrR*) (Fig. 10) confirmed our initial observations and suggests an essential role for QsrR quinone-mediated gene regulation in the PDI response.

The increased expression of SAOUHSC_00093, encoding a manganese (Mn)-dependent superoxide dismutase (SodM), illustrates another mechanism that works synergistically with the quinone detoxification regulon to resist oxidative stress in the PDI-tolerant population. In an infection, S. aureus expresses two SODs, SodA and SodM, which utilize either Fe or Mn as a cofactor and enable the bacterium to overcome the oxidative stress from neutrophils and other immune cells. As the infection progresses, the host sequesters Mn from the infection site, resulting in an Mn-depleted environment and a transition in SodA-SodM activity. Recent work by Garcia et al. (38) has shown that SodA protects S. aureus from oxidative stress in Mn-replete environments, whereas SodM is critical for protecting S. aureus from oxidative stress in Mn-deplete environments where it utilizes Fe as a cofactor. The increased expression of the iron transport siderophores SirA and SirB demonstrates a synergistic adaption of S. aureus metabolic and SodM-mediated oxidative response functions for PDI tolerance.

The success of S. aureus in pathogenesis has been attributed to its sophisticated signaling systems, including the global accessory gene regulator (*agr*) that senses and responds to environmental and host signals with adaptive strategies that are required for a successful infection (39–45). Previous studies with *agr* have yielded conflicting results on its role in PDI susceptibility, with the disparities in the results apparently being dependent on the genotype of the S. aureus strains and the structure of the photosensitizers (15, 26–28). A recent study used S. aureus SH1000, a strain derived from S. aureus 8325-4 by successive cycles of UV irradiation, which introduces cryptic mutations into the genome with unknown effects (18). We, therefore, tested HG003*agr*::*tetM* for its ability to develop resistance to PDI and observed that, despite the Agr-null phenotype, this strain was still able to develop resistance to PDI (Fig. S3). We also note that expression of *agr* was not increased in our transcriptome analysis of the PDI-tolerant population (Table S2). We recognize that the conflicting results with *agr* and other factors that influence the PDI response are due not only to variation in the genotypes of S. aureus strains but also to experimental variables in the PDI treatment. Two types of reactions occur in PDI; a type I reaction, which generates ROS such as superoxide (^·^O_2_^−^), hydroxyl radical (^·^OH), and hydrogen peroxide (H_2_O_2_), or a type II reaction, which produces singlet oxygen (^1^O_2_^−^). Type I and type II reaction products can be produced simultaneously in PDI, with the proportion of each being dependent on the type of photosensitizer used and the ionic strength of its solvent (46, 47). We observed that HG003 appears to be less sensitive to PDI when water is used as a solvent than when phosphate-buffered saline (PBS) is used, needing 16 times the amount of MB to achieve a comparable log reduction in CFU (data not shown). This finding and the variability between our study and others comparing PDI and PDI tolerance raise important concerns on the potential of PDI to treat human infections. Future development of PDI will require standardization of treatment conditions, like that used for MIC testing of antibiotics.

In conclusion, we have demonstrated that repeated exposure of S. aureus HG003 and isogenic mutant MMR and *agr* strains to PDI leads to PDI tolerance. The tolerance to PDI was maintained upon repeated nonselective subcultures with no additional PDI treatment. Genome-wide transcriptome analyses revealed numerous pathways in DNA repair, detoxification, and the response to oxidative stress, efflux transporters, and metabolism that contributed to the initial PDI response, with unique functions in iron transport, metabolism, and oxidative enzymes, and a central role of the MHQ-quinone regulon that contribute to PDI tolerance. Comparative genome analysis of HG003 revealed no adaptive, heritable mutations in surviving bacteria exposed to a single PDI treatment or to the methylene blue photosensitizer alone. A single nonsynonymous SNP was found in the region corresponding to the quinone binding pocket of the quinone response regulator, QsrR, in the HG003 genome of the PDI tolerant population. Increased PDI tolerance of a *qsrR* gene deletion strain suggest an essential role for QsrR-quinone-mediated gene regulation in the PDI response.

## MATERIALS AND METHODS

### Selection of S. aureus strains for PDI.

The susceptibility of S. aureus to PDI is strain dependent, suggesting that predisposing genetic adaptations to PDI exist within laboratory or infecting S. aureus isolates (48). To eliminate the confounding influence of S. aureus genetic variability observed in previous PDI studies, we employed the S. aureus model strain HG003 and isogenic HG003 mutants for the work presented here. S. aureus NCTC8325, the parental strain of HG003, is the progenitor of a large series of derivative strains that have been used for the development of molecular tools and the study of genetic regulation, virulence factors, host responses, and interaction with the immune system in the S. aureus research community (18). With the exception of two regulatory pathways, RsbU and TcaR, all global regulators in NCTC8325, including the accessory gene regulator (*agr*) system, are functional. Both RsbU, a significant element in the S. aureus regulation of staphyloxanthin synthesis and membrane fluidity, and TcaR, a cell wall-associated regulator of protein A, have been repaired and are functional in HG003. Because HG003 is a well-characterized strain and possesses all known regulatory and physiological functions that contribute to the multifactorial response to PDI, it is a potential reference strain for use in all PDI studies (18). S. aureus ATCC 25923, a strain that has previously been reported not to develop tolerance to PDI (22), was included in our study as a comparative control for the response of HG003 to PDI. All bacterial strains are available from the corresponding author.

### Strains and materials.

S. aureus strains HG003, HG003Δ*mutSL*, HG003Δ*mutY*, HG003*agr*::*tetM* (8, 18), ATCC 25923 (49), HG003Δ*qsrR*, and HG003Δ*qsrR* p-*qsrR* were used in this study (Table 1). Bacterial cells were inoculated as single colonies from tryptic soy agar (TSA) into tryptic soy broth (TSB) and cultured aerobically at 250 rpm and 37°C to stationary phase. The photosensitizers methylene blue (MB; Akorn, Inc., Lake Forest, IL) and toluidine blue O (TBO; Sigma-Aldrich, St. Louis, MO) were protected from light until use. For PDI treatments, bacterial cells were irradiated at a fluence of 2.4 J cm^−2^ of visible light from a 48-cm by 48-cm light box equipped with a bank of fluorescent lamps (Sylvania Gro-Lux; 15 W; model F15T8/GRO). The irradiance at the surface of the light source was 4.0 mW cm^−2^, and the spectrum of light was such that approximately 67% of the power was emitted within the 575- to 700-nm range (50).

**TABLE 1:** S. aureus strains used in this study

S. aureus strain	Description^*a*^	Reference
HG003	Laboratory strain	18
HG003 G1D1	1× MB−L−	This study
HG003 G1D3	3× MB−L−	This study
HG003 G1D7	7× MB−L−	This study
HG003 G3D1	1× MB+L−	This study
HG003 G3D3	3× MB+L−	This study
HG003 G3D7	7× MB+L−	This study
HG003 G4D1	1× MB+L+ (PDI)	This study
HG003 G4D3	3× MB+L+ (PDI)	This study
HG003 G4D7	7× MB+L+ (PDI)	This study
HG003Δ*mutY*	Hypermutator strain	8
HG003Δ*mutSL*	Hypermutator strain	8
HG003*agr*::*tetM*	*agr* replaced with *tetM*	This study
HG003Δ*qsrR*	*qsrR* allelic deletion strain	This study
HG003Δ*qsrR* pCN40erm-*qsrR*	*qsrR* allelic deletion strain carrying *qsrR* complement	This study
ATCC 25923	Laboratory strain	49

a The number (e.g., 1×) indicates the number of consecutive days of treatment. MB−L−, passaged without PDI; MB+L−, treated with MB only; MB+L+, treated with MB and light (PDI).

### Construction of S. aureus HG003Δ*qsrR* and complemented strains.

The Escherichia coli-S. aureus shuttle vector pWedge was used in the construction of an in-frame deletion of *qsrR* in S. aureus HG003 (HG003Δ*qsrR*) by allelic exchange as previously described using synthesized gene blocks spanning positions from nucleotide 2187037 to 2186538 and from nucleotide 2186201 to 2185702 of the S. aureus NCTC 8325 genome (GenBank no. CP000253.1) in SAOUHSC_02364 (*qsrR*) (8). The deletion was confirmed by targeted sequencing. Gene complement constructs were created by ligating the full-length *qsrR* gene into pCN40 (HG003Δ*qsrR* p-*qsrR*) (51).

### Initial PDI treatment.

Stationary-phase cells were washed twice in PBS and adjusted to an optical density at 600 nm (OD_600_) of 1.0 in PBS (approximately 7 × 10^8^ CFU/ml). The cell suspension was then divided into two aliquots, one treated with 12.5 μg ml^−1^ (33 μM) methylene blue (MB+) and one left untreated (MB−). This concentration was chosen as it resulted in a 3- to 4-log reduction in CFU but allowed selection of surviving cells. Cells were incubated at room temperature for 30 min with periodic vortexing. After incubation with MB, cells were pelleted and resuspended in equal volumes of PBS. Cell suspensions (7 × 10^8^ CFU) were then added in duplicate to two 12-well tissue culture dishes (1 ml/well). One dish was exposed to broadband visible light (L+) and the other shielded from light (L−) for 10 min. Cells from each treatment group (MB−L−, MB−L+, MB+L−, and MB+L+) were serially diluted and plated on TSA. Plates were incubated overnight at 37°C, and CFU were enumerated. The general protocol for PDI treatment is illustrated in Fig. 1.

### Successive PDI treatments.

Immediately following the PDI treatment described above, an aliquot from each treatment group was diluted 1:200 into 5 ml of fresh TSB (total of 1.75 × 10^7^ CFU) and cultured overnight for a subsequent round of PDI. In addition to the sequentially treated cells, each day a naive culture (no previous PDI exposure) was inoculated from TSA to serve as a control. This procedure was repeated for a maximum of 8 consecutive days, with cells from the initial PDI treatment group being sequentially treated on 7 additional consecutive days. In this paper, “naive culture” refers to a culture that was started from a single isolated colony and was not subcultured or exposed to MB−L−, MB+L−, MB−L+, or MB+L+ (PDI).

### MB quantitation.

Cultures of naive, 7-day sequentially treated MB+L− and 7-day sequentially treated MB+L+ groups were treated with MB as in previous experiments. After incubation, cells were pelleted by centrifugation, aliquots of the supernatants were saved, and bacterial pellets were resuspended in 5 ml of PBS. MB concentration was quantitated in both the supernatant and cell pellet from the peak amplitude of absorbance measurements via spectrophotometry (Varian 50 Bio spectrophotometer; Varian, Palo Alto, CA) based on a calibration curve of known MB concentrations.

### PDI treatment of passaged cells with TBO.

Naive, 7-day sequentially treated MB+L−, and 7-day sequentially treated MB+L+ cells were cultured as described previously and subjected to a single PDI treatment as detailed for MB, except that cells were treated with 3.13 μg ml^−1^ (10.2 μM) TBO. Preliminary experiments demonstrated that this concentration resulted in a similar reduction in CFU as treatment with 12.5 μg ml^−1^ (33 μM) of MB. Following PDI, cells were serially diluted 10-fold and plated on TSA for CFU determination.

### Determination of heritability of PDI resistance.

Naive and 7-day sequentially treated MB+L+ cells were cultured in TSB for 24 h. These cultures were then diluted 1:200 into fresh TSB and subcultured daily without treatment for 3 days. On the third day, fresh naive and 7-day sequentially treated MB+L+ cultures were inoculated in parallel with the 3-day sequentially subcultured groups to serve as nonsubcultured controls. PDI was then performed on all groups in the same experiment as described previously.

### RNA-Seq transcriptome analysis.

Whole transcriptome analysis (RNA-Seq) was used to evaluate four groups of PDI-susceptible and PDI-resistant populations. Groups are defined as follows: group 1 is naive HG003 cultures that were not subcultured or exposed to MB−L−, MB+L−, MB−L+, or MB+L+ treatments; group 2 received seven sequential daily MB−L− treatments, followed by a single MB+L+ (PDI) treatment; group 3 received seven sequential daily MB+L− treatments, followed by a single MB+L− treatment; group 4 received seven sequential daily MB+L+ treatments, followed by a single MB+L+ treatment (PDI). All groups were compared to a non-PDI-treated control, and experiments were performed in biological duplicate. Immediately following PDI treatment, the bacterial cultures were placed in RNAprotect (Qiagen, Valencia, CA) to stabilize RNA. RNA was isolated by mechanical lysis in TRIzol using lysing matrix B tubes and MP FastPrep-24 (MP Biomedicals, Solon, OH) and purified using DirectZol columns (Zymo Research, Irvine, CA). Residual DNA was removed using Turbo DNase (Thermo Fisher Scientific, Waltham, MA).

cDNA libraries were constructed using the TruSeq Stranded mRNA library kit (Illumina, San Diego, CA), analyzed for quantity and quality, and sequenced on an Illumina HiSeq 2500 instrument (Illumina, San Diego, CA). Sequencing reads were cleaned using the Trimmomatic-0.32 workflow prior to being mapped to the S. aureus NCTC8325 genome with STAR-2.4.2a. Cufflinks2.0.2 was then used with NCTC8325 gene annotations to analyze differential gene expression with a 95% confidence interval (and a false discovery rate [FDR] of 0.05). RNA expression levels for each gene were expressed as fragments per kilobase per million reads mapped (FPKM). Genes for which all samples did not have at least an FPKM value of 1 were removed from consideration before downstream analysis. A greater-than-log_2_-fold increase or decrease in expression level was used to identify genes expressed at significantly different levels between experimental groups. A *P* value cutoff of 0.05 was set to identify genes that were significantly different in expression between group 1 and groups 2, 3, and 4. The R packages DESeq2_1.10.1 and pheatmap_1.0.8 were used to create figures of these data. PATRIC (52) was used in subsequent pathway and functional COG (clusters of orthologous groups) analysis. Principal-component analysis and visualization were performed using R version 3.3.3 on the arcsin square-root-transformed relative abundances of all pathways present.

### qRT-PCR.

Two independent replicates of the RNA used for RNA-Seq were converted to cDNA using the iScript cDNA synthesis kit (Bio-Rad, Hercules, CA). Quantitative real-time PCRs (qRT-PCRs) were carried out on an AB Applied Biosystems GeneAmp PCR system 9700 (Applied Biosystems, Foster City, CA) using iQ SYBR green Supermix (Bio-Rad). The primers listed in Table S1 were used for analysis with the following qRT-PCR parameters: one 3-min incubation at 95°C, followed by 40 cycles of 10 s at 95°C and 30 s at 62°C. Melting curves from 65°C to 95°C with 0.5°C increments for 5 s were performed at the end of each cycle. Runs were performed in triplicate, and transcript levels were obtained based on absolute copy numbers from standard curves generated from genomic DNA as described previously (53).

10.1128/mSphere.00762-20.5 TABLE S1 Primers used for qRT-PCR in this study. Download Table S1, DOCX file, 0.01 MB. Copyright © 2021 Snell et al. 2021 Snell et al. This content is distributed under the terms of the Creative Commons Attribution 4.0 International license.

10.1128/mSphere.00762-20.6 TABLE S2 Summary of transcriptome comparisons between group 1 (reference) and groups 2, 3, and 4. Download Table S2, XLSX file, 0.1 MB. Copyright © 2021 Snell et al. 2021 Snell et al. This content is distributed under the terms of the Creative Commons Attribution 4.0 International license.

10.1128/mSphere.00762-20.7 TABLE S3 Summary of single nucleotide polymorphisms (SNPs) in genomes of isolates from treatment groups 1, 3, and 4 at days 1, 3, and 7. Download Table S3, XLSX file, 0.01 MB. Copyright © 2021 Snell et al. 2021 Snell et al. This content is distributed under the terms of the Creative Commons Attribution 4.0 International license.

### Genomic sequencing and nucleotide variant (SNP/indel) analysis of PDI-susceptible and PDI-tolerant HG003.

Whole-genome analysis was used to determine genetic differences in PDI-susceptible and PDI-resistant populations of HG003. Bacterial cells were disrupted using lysostaphin (AMBI Products LLC, Lawrence, NY), and genomic DNA was extracted and purified using DNA blood and tissue kit (Qiagen, Valencia, CA). DNA libraries were prepared using NexteraXT (Illumina, San Diego, CA), analyzed for quantity and quality, and subjected to paired-end sequencing on an Illumina HiSeq 2500 instrument (Illumina, San Diego, CA). Sequencing reads were demultiplexed using bcltofastq-1.8.4 and cleaned with Trimmomatic-0.32 prior to being assembled using SPAdes v3.14.1 and aligned to the S. aureus NCTC8325 genome using ParSNP v1.2 from the Harvest suite to identify single nucleotide polymorphisms (SNPs) and insertions/deletions (indels) (54, 55).

### PDI treatment of *qsrR* allelic deletion (HG003Δ*qsrR*) and *qsrR* allelic deletion complemented (HG003Δ*qsrR* p-*qsrR*) strains.

Naive HG003, 7-day sequentially treated HG003 MB+L+, naive HG003Δ*qsrR*, and naive HG003Δ*qsrR* p-*qsrR* cells were cultured in TSB for 24 h. The cells were washed twice in PBS, adjusted to an OD_600_ of 1.0 in PBS (approximately 7 × 10^8^ CFU/ml), and treated with MB−L−, MB−L+, MB+L−, and MB+L+ as described above in “Initial PDI treatment.”

### Data availability.

All RNA-Seq sequence reads, differential gene expression (DGE) values, the assembled draft genomes, and raw sequence reads are publicly available at NCBI and SRA under NCBI BioProject ID PRJNA644790.
