# Supplementary figures and images for: Staphylococcus aureus Tolerance and Genomic Response to Photodynamic Inactivation
Source: mSphere. 2021 Jan 6;6(1):e00762-20. doi: 10.1128/mSphere.00762-20 (PMC7845598; doi:10.1128/mSphere.00762-20)

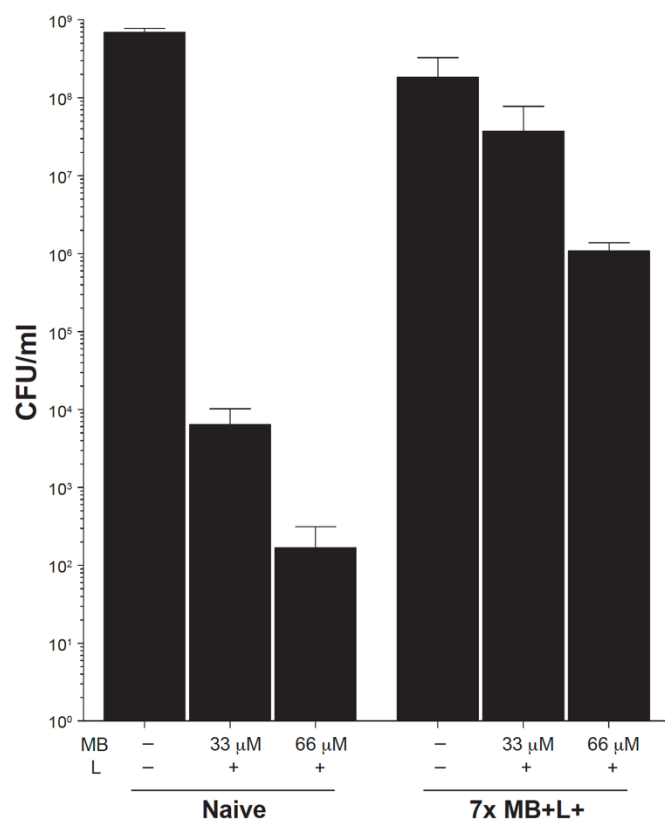

**Figure S1. PDI of resistant cells with 2x MB concentration.**

Supplement: FIG S1 [file mSphere.00762-20_sf001.pdf]

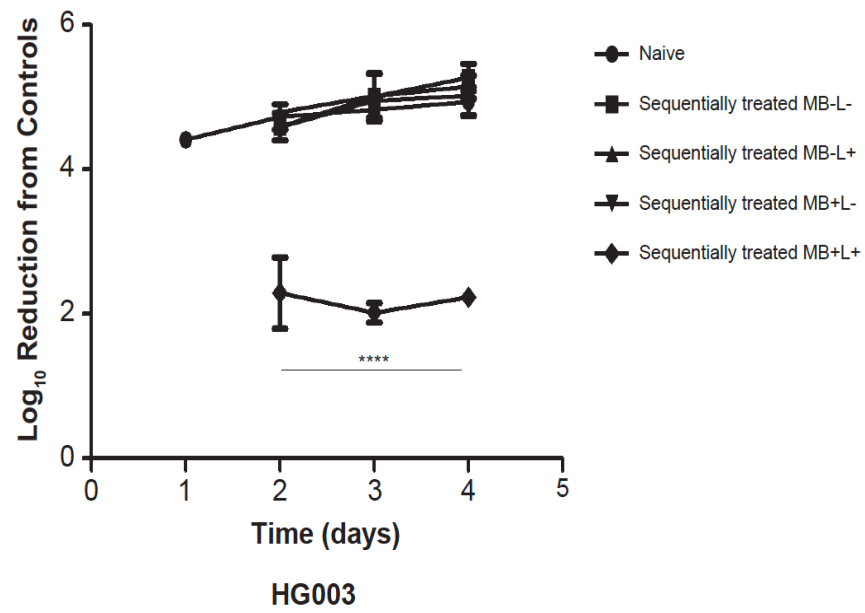

**Figure S2. PDI resistance study without wash step.**

Supplement: FIG S2 [file mSphere.00762-20_sf002.pdf]

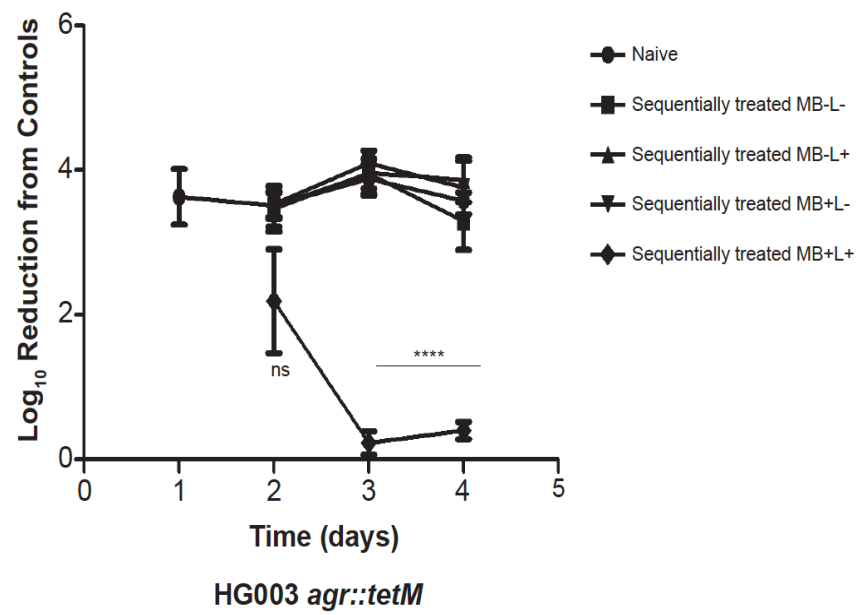

**Figure S3. PDI resistance develops in *S. aureus* HG003 *agr::tetM*.**

Supplement: FIG S3 [file mSphere.00762-20_sf003.pdf]

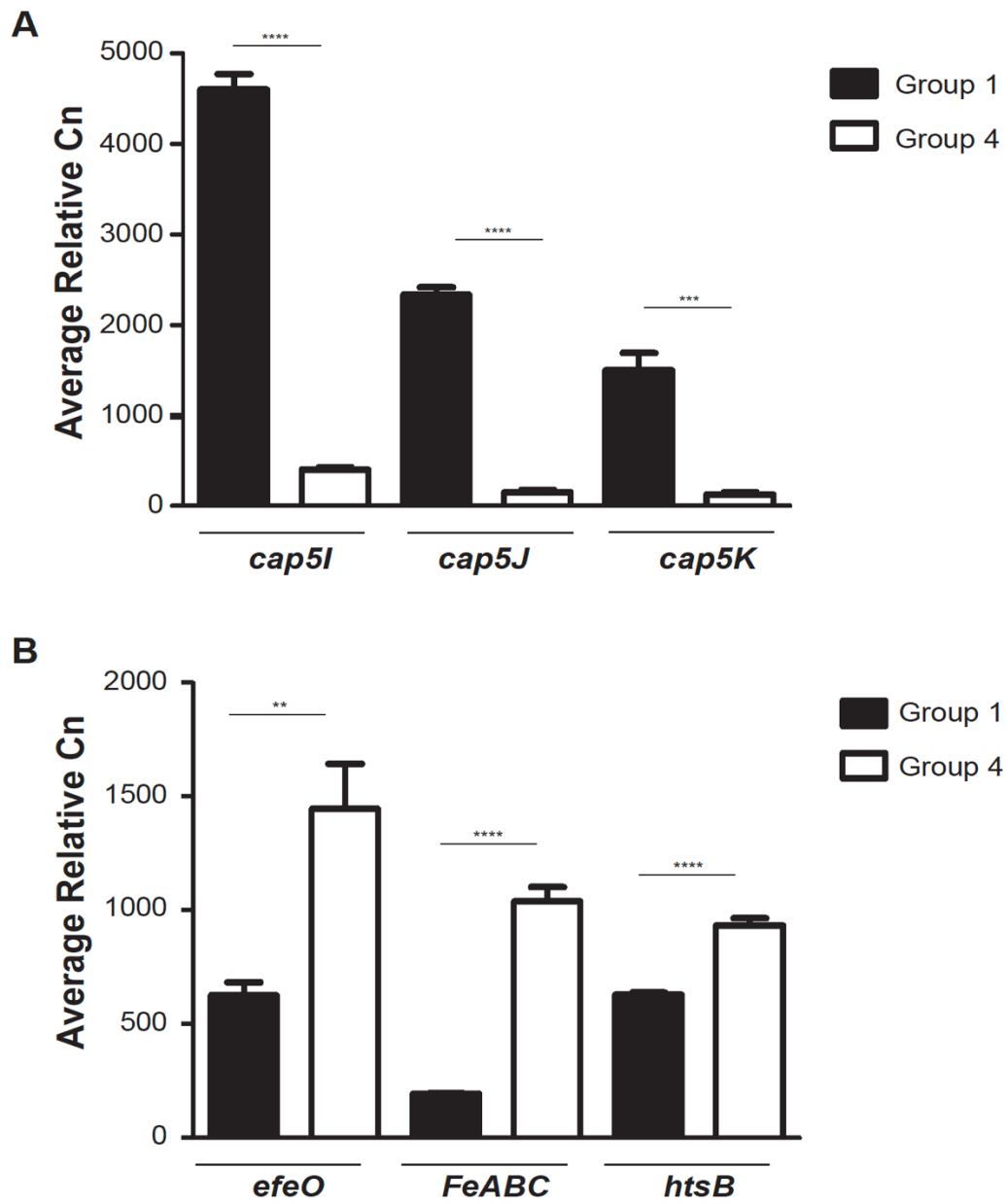

**Figure S4. qRT-PCR indicate similar trends in differential expression of select genes.**

Supplement: FIG S4 [file mSphere.00762-20_sf004.pdf]
