# Supplementary material for: Staphylococcus aureus Tolerance and Genomic Response to Photodynamic Inactivation
Source: mSphere. 2021 Jan 6;6(1):e00762-20. doi: 10.1128/mSphere.00762-20 (PMC7845598; doi:10.1128/mSphere.00762-20)
Supplement: TABLE S1 [file mSphere.00762-20_st001.docx]

| **Primer/Probe** | **Sequence 5'-3'** | **Application** |
| --- | --- | --- |
| Fe ABC Fwd | ACGGTAGAGCAGTTGGTAAAC | qRT-PCR Validation |
| Fe ABC Rev | GCAACAAATCTAGCGCATCAT | qRT-PCR Validation |
| Cap5I Fwd | CAAGGTCAAATTGTAATGGACAGAG | qRT-PCR Validation |
| Cap5I Rev | CGGACCAAACCCTCGAATTAT | qRT-PCR Validation |
| Cap5J Fwd | GCTTCGTACCTTTCAGCATTTAC | qRT-PCR Validation |
| Cap5J Rev | GCCCTCCTGGTATAAACACAATA | qRT-PCR Validation |
| Cap5K Fwd | AGGCGAGTATCAAAGTGAAGAT | qRT-PCR Validation |
| Cap5K Rev | GATATAGTTACAGCTGTTCCACCTA | qRT-PCR Validation |
| HemeABC (HtsB) Fwd | GACACCGACATACCTGTAAGAA | qRT-PCR Validation |
| HemeABC (HtsB) Rev | CGATAATGGAACTAGGCGATGA | qRT-PCR Validation |
| EfeO Fwd | AGTGATGATATCCAAATGAACTTCG | qRT-PCR Validation |
| EfeO Rev | GCAAATGCTTTACGGTCTTTCT | qRT-PCR Validation |

**Table S1.** **Primers used for qRT-PCR in this study.**
